# Thermal behavior and microstructures of cathodes for liquid electrolyte-based lithium batteries

**DOI:** 10.1038/s41598-018-34017-2

**Published:** 2018-10-23

**Authors:** Hirofumi Tsukasaki, Wataru Fukuda, Hideyuki Morimoto, Toshihiro Arai, Shigeo Mori, Akitoshi Hayashi, Masahiro Tatsumisago

**Affiliations:** 1 Department of Materials Science, Graduate School of Engineering, Osaka Prefecture University, 1-1, Gakuen-cho, Naka-ku, Sakai, Osaka 599-8531 Japan; 2 Graduate School of Science and Technology, Gunma University, Kiryu, Gunma, 376-8515 Japan; 3 Department of Applied Chemistry, Graduate School of Engineering, Osaka Prefecture University, 1-1, Gakuen-cho, Naka-ku, Sakai, Osaka 599-8531 Japan

## Abstract

Lithium-ion batteries are widely used as a power source for portable equipment. However, the use of highly flammable organic solvents in the liquid electrolyte component in these batteries presents a serious safety concern. In this study, the thermal stability of battery cathodes comprising LiNi_1/3_Mn_1/3_Co_1/3_O_2_ (NMC) and LiPF_6_-based electrolyte solutions have been investigated using transmission electron microscopy (TEM) and differential scanning calorimetry (DSC) methods. *Ex situ* TEM measurements revealed that significant structural change occurred in the charged NMC composite after heating at a temperature above the exothermal peaks. It was found that LiF nanocrystallites precipitated in LiPF_6_ and that a number of nanoscale stacking faults developed in the $R\bar{3}m$ layered structure of NMC. The results suggested that the decomposition reaction of LiPF_6_ and the structural change of NMC were directly associated with the exothermic reaction in the liquid electrolyte-based NMC electrode composite.

## Introduction

Lithium-ion batteries exhibiting excellent cycling characteristics and a high energy density are currently used as a power source for portable equipment. Recently, large-sized lithium-ion batteries with a higher energy density have become very promising candidates for use in electric vehicles, hybrid electric vehicles, and accumulators for household use. However, because highly flammable organic solvents are used in liquid electrolytes, there are serious safety concerns, such as the possibility of thermal runaway^1–4^. To realize large-sized lithium cells with a higher energy density and excellent cycling performance, it is thus extremely important to clarify the mechanism of the exothermic reaction.

In general, liquid electrolytes are composed of organic solvents such as ethylene carbonate (EC) and ethyl methyl carbonate (EMC), in which the lithium hexafluorophosphate salt (LiPF_6_) is dissolved. As for the cathode materials, layered oxides Li*M*O_2_ (M: Co, Ni, Mn, Al), spinel oxides LiMn_2_O_4_ and lithium iron phosphate LiFePO_4_ are commonly used^5–11^. Thus far, it has been proposed that the thermal stability of these cathode materials and the LiPF_6_ electrolyte is directly involved in the exothermic reaction. The charged cathode materials are chemically unstable. For example, delithiated Li*M*O_2_ transforms into a spinel Li_1−x_*M*_2_O_4_ phase and decomposes into *M*_3_O_4_ or *M*O-type rock salt crystal phases, accompanied by an increase in the reaction temperature^12–15^. It is well known that such structural changes during heating are accompanied by the oxygen release. Any O_2_ released reacts with the organic solvent, leading to a large exothermic reaction^16–19^. In contrast, LiPF_6_ decomposes upon heating and exhibits exothermic reactions even without the presence of anode and cathode materials. Moreover, the decomposition reaction of LiPF_6_ leads to the generation of PF_5_ gas, which could react with EC and DMC solvents at room temperature^20,21^. However, despite the possibilities of these factors, the exact origin of the exothermic reactions in liquid electrolyte-based lithium cells is still unknown.

This study investigated the thermal stability of cathode composites in a liquid electrolyte-based cells using transmission electron microscopy (TEM) and differential scanning calorimetry (DSC) methods. LiNi_1/3_Mn_1/3_Co_1/3_O_2_ (NMC) was used as a cathode material, while 1 M (M: mol L^−1^) LiPF_6_-EC/EMC (mixing volume ratio = 3:7) was used as a base electrolyte solution. After the thermal behavior of the NMC composite was examined via DSC measurements, *ex situ* TEM observations were conducted at room temperature. On the basis of structural changes caused by heat treatment, the origin of the exothermic reactions in the charged NMC composite was discussed.

## Results and Discussion

**Figure 1:**
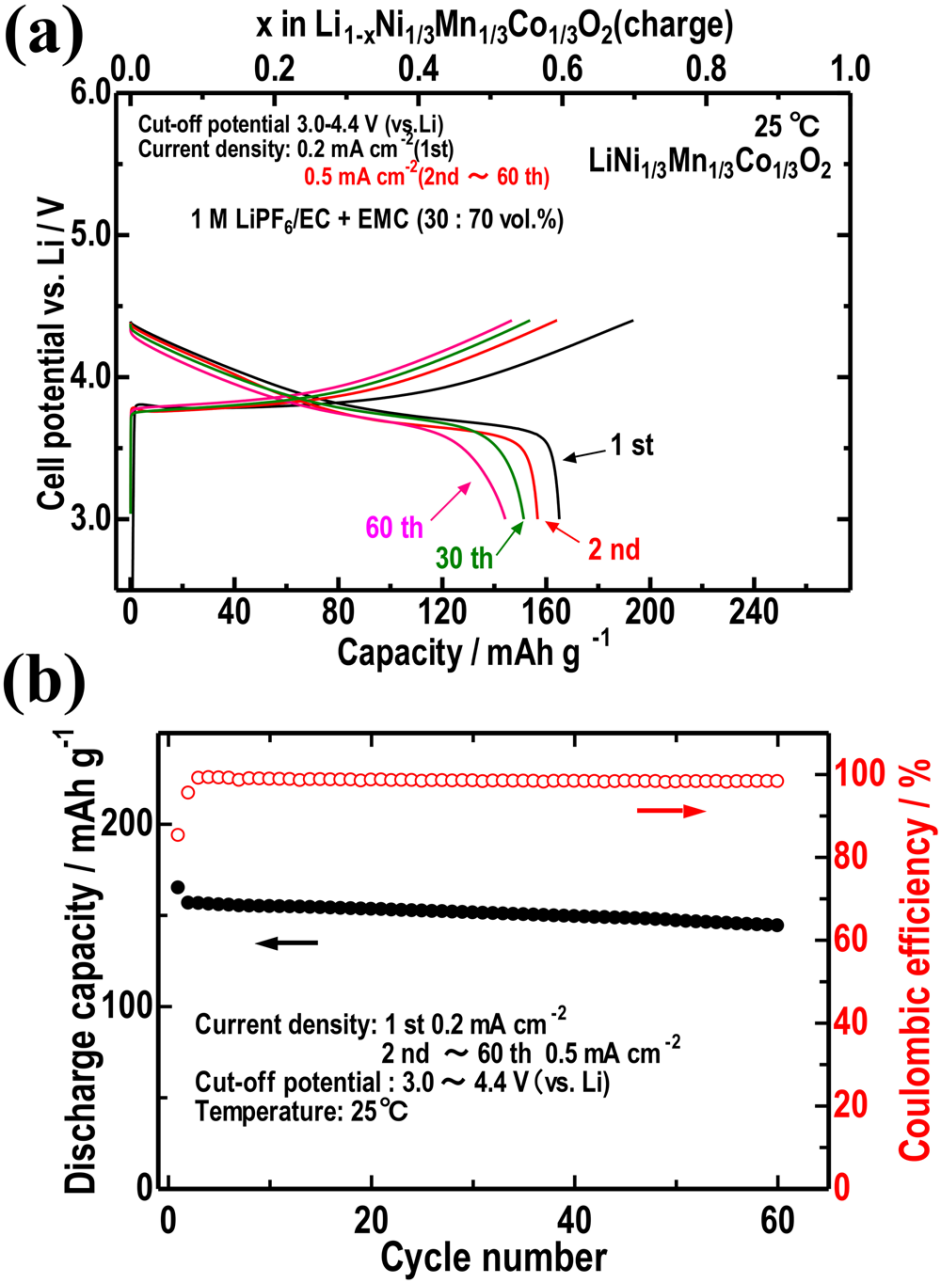
(**a**) Charge-discharge curves of the Li/LiNi_1/3_Mn_1/3_Co_1/3_O_2_ cell at 25 °C. (**b**) Long-term cycling performance of the cell.

**Figure 2:**
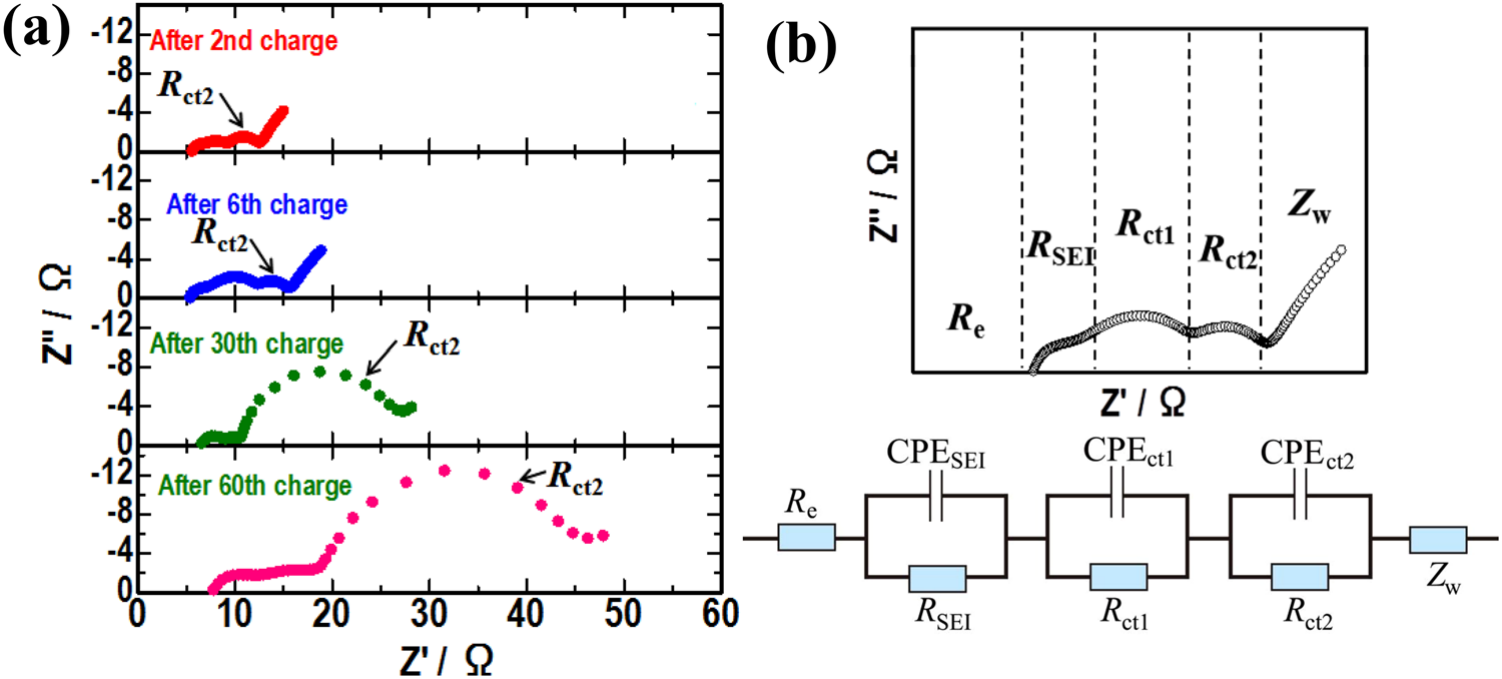
(**a**) Nyquist plots of the cell after the 2nd, 6th, 30th and 60th charge cycles. (**b**) The equivalent circuit was conducted in order to fit the Nyquist plots.

Figure 1(a) shows the initial, 2nd, 30th and 60th charge–discharge curves of the fabricated cells using NMC with 1 M LiPF_6_ (EC/EMC = 30:70 vol.%) as the electrolytes. The current density of the initial charge-discharge process was 0.2 mA cm^−2^. After the 2nd charge–discharge, the current density was 0.5 mA cm^−2^. To examine charge-discharge cycle performance in a relatively high voltage, in this study, the cells were operated at 25 °C in the voltage range of 3.0–4.4 V (vs. Li). Figure 1(b) shows the corresponding cycling performance of the cells. Based on the charge-discharge properties shown in Fig. 1(a,b), the capacity gradually decreases as the number of charge-discharge cycles increases. To clarify the factors behind the decline in capacity, electrochemical impedance measurements were conducted. Figure 2(a) shows Nyquist plots of the cells after the 2nd, 6th, 30th and 60th charge cycles. Five impedance components were detected in the impedance plots, which could be explained by the equivalent circuit, as shown in Fig. 2(b)^22,23^. The *R*_e_ value in the high-frequency region can be attributed to electrolyte resistance. The *R*_SEI_, *R*_ct1_ and *R*_ct2_ value in the medium-frequency region represent the solid electrolyte interface resistance, charge transfer resistance of the Li/electrolyte and charge transfer resistance of the NMC/electrolyte, respectively. The *Z*_w_ value in the low-frequency region denotes the Warburg impedance. As the number of charge cycles increased, the resistance component *R*_ct2_ significantly increased, as shown in Fig. 2(a). This suggests that capacity decline can be fundamentally attributed to the increase in the charge transfer resistance of the NMC/LiPF_6_ electrolyte.

**Figure 3:**
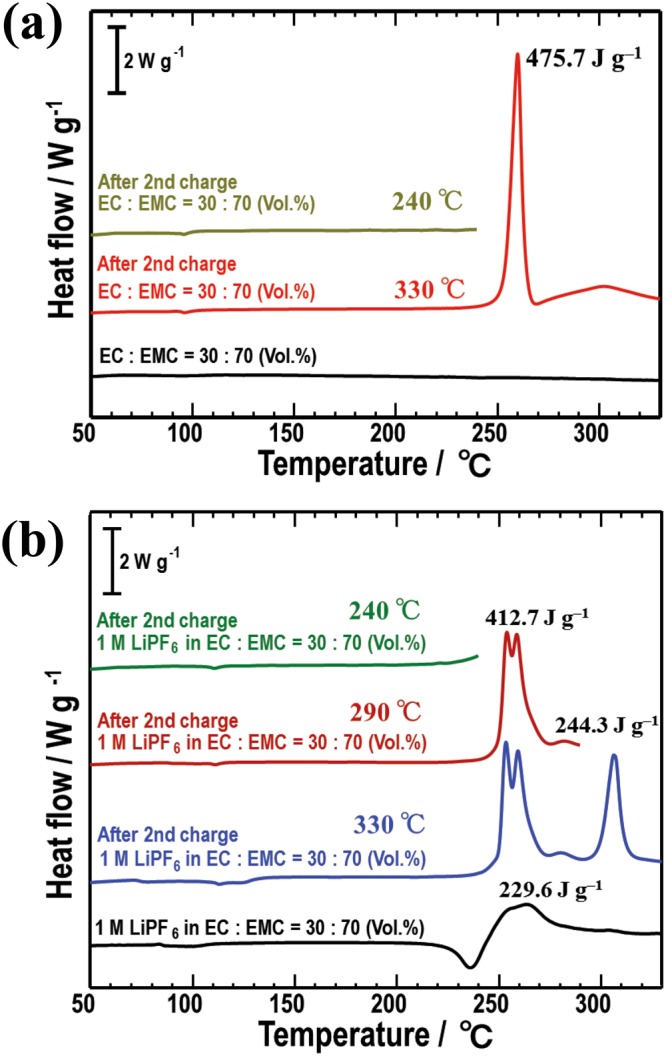
The DSC curves of the NMC composites after the 2nd charge cycles in the presence of (**a**) only the EC/EMC solution and (**b**) the 1 M LiPF_6_-EC/EMC solution. For reference, the DSC curves of only EC/EMC solvent and only LiPF_6_ and EC/EMC solvent are shown at the bottom in (**a**,**b**), respectively.

To investigate the thermal behavior, DSC measurements were then performed for two types of NMC composites after the 2nd charge: one containing only an EC/EMC solution, the other containing a 1 M LiPF_6_-EC/EMC solution. Figure 3(a) shows the DSC curves of the two charged NMC composites in the presence of the EC/EMC solution over a temperature range of room temperature to 240 °C and 330 °C, respectively. For reference, the DSC curve of only the EC/EMC solvent is also shown at the bottom. The sample heated at 330 °C underwent a large exothermic reaction, showing a heating value of approximately 475.7 J g^−1^ at 260 °C. In contrast, the sample heated at 240 °C showed no exothermic reaction. Because no exothermic reactions could be observed in the DSC curve of only the EC/EMC solvent, the exothermic peak at 260 °C should be derived from not organic solvents but NMC and/or electrolyte solution. Figure 3(b) shows the DSC curves of the NMC composites in the presence of a 1 M LiPF_6_-EC/EMC solution. Three charged NMC composites were prepared and were heated from room temperature to 240 °C, 290 °C and 330 °C, respectively. For reference, the DSC curve of only LiPF_6_ and the EC/EMC solvent is also shown at the bottom. In the case of the EC/EMC solution containing LiPF_6_, the sample heated at 330 °C exhibited two exothermic reactions at approximately 255 °C and 306 °C. The heating values of the exothermic peaks at 255 °C and 306 °C were 412.7 J g^−1^ and 244.3 J g^−1^, respectively. This thermal behavior is significantly different from that in the case of using only the EC/EMC solution. The sample heated at 240 °C exhibited no exothermic reactions and the sample heated at 290 °C exhibited one exothermic reaction at 255 °C. In addition, the DSC curve of only LiPF_6_ and the EC/EMC solvent exhibited an exothermic peak at 255 °C with the heating values of 229.6 J g^−1^. Since the EC/EMC solvent itself is not involved in exothermic reactions, as proved in Fig. 3(a), the exothermic peak at 255 °C should be derived from LiPF_6_. To clarify the detailed origin of each exothermic peak observed in Fig. 3(a,b), the microstructures of the charged NMC composites after the different heating temperatures were examined using *ex situ* TEM at room temperature.

**Figure 4:**
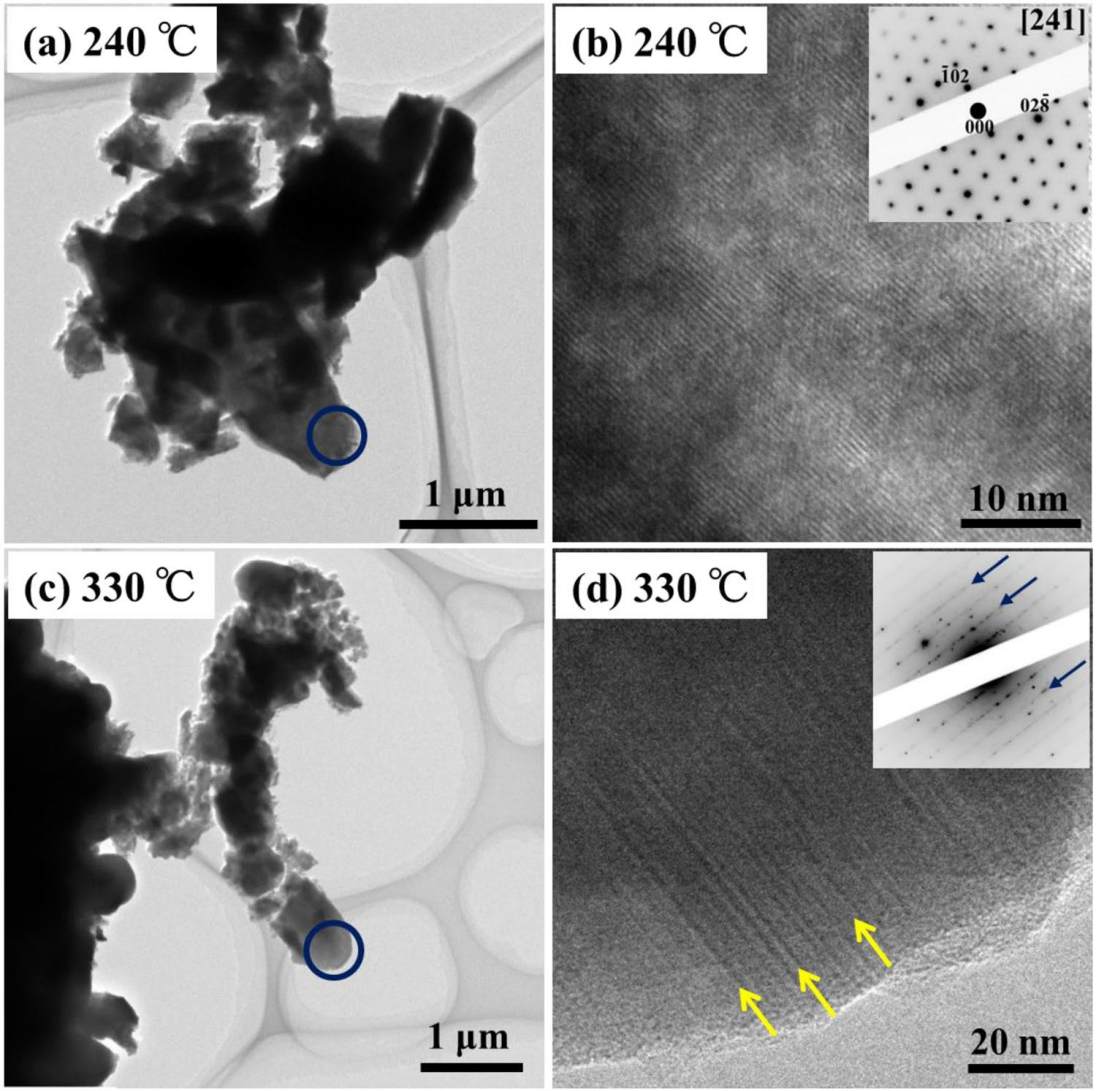
*Ex situ* TEM measurements of the charged NMC composites with the EC/EMC solution. The BF images in (**a**,**c**) show the morphology of the NMC composites after heating at 240 °C and 330 °C, respectively. The HR images in (**b**,**d**) show the microstructure in NMC after heating at 240 °C and 330 °C, respectively.

Figure 4 shows the *ex situ* TEM observation results for the NMC composites after the 2nd charge cycles in the presence of the EC/EMC solution. The bright field (BF) images in (a) and (c) show the morphology of the NMC composites after heating at 240 °C and 330 °C, respectively. It should be noted here that the EC and EMC solvents were volatilized by heating during the DSC measurements. The SEM observations showed that NMC tended to be microparticulated after heating at 330 °C (see Supplementary Fig. S1). The high-resolution (HR) images in (b) and (d) show the microstructure in NMC after heating at 240 °C and 330 °C, respectively. These images were obtained from the regions indicated by the blue circles in (a) and (c). The corresponding electron diffraction (ED) patterns are shown in the insets. After heating at 240 °C, lattice fringes corresponding to the [241]-zone axis ED patterns derived from the *R*$\bar{3}$*m* layered structure were clearly observed for the NMC particles, as shown in Fig. 4(b). After heating at 330 °C, streaks appeared in the ED pattern and the corresponding nanoscale linear contrasts could be seen, as indicated by the arrows in Fig. 4(d). These linear contrasts and streaks indicated the presence of stacking faults. That is, when the temperature exceeded 260 °C, NMC particles were microparticulated and a number of stacking faults were introduced into the initial *R*$\bar{3}$*m* layered structure.

**Figure 5:**
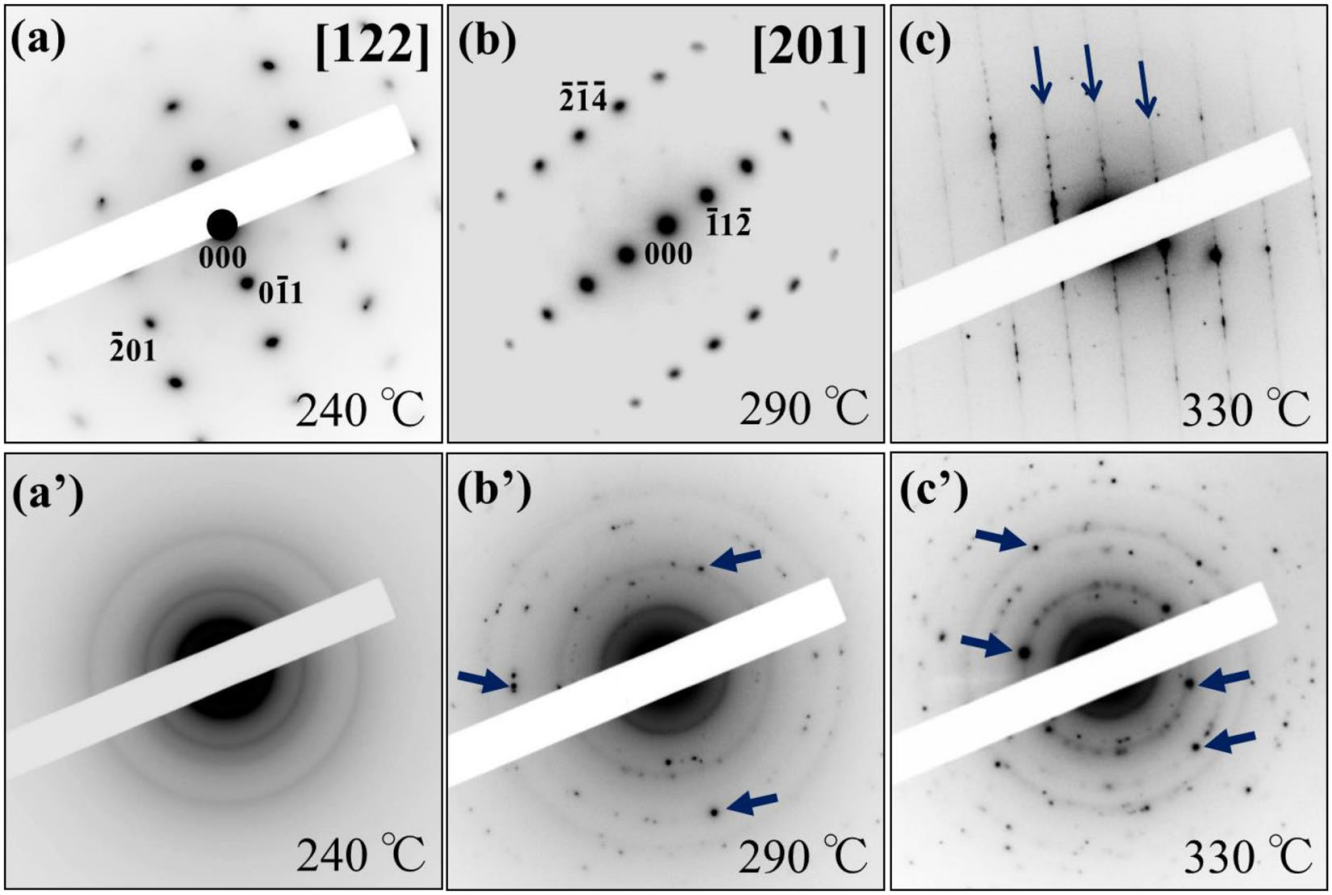
The heat temperature dependence of the ED patterns in the charged NMC composites with the 1 M LiPF_6_-EC/EMC solution. The ED patterns of NMC are shown in (**a**–**c**), while the ED patterns of LiPF_6_ are shown in (**a’**), (**b’**) and (**c’**).

The same analysis was conducted for the NMC composites after the 2nd charge cycle in the presence of the 1 M LiPF_6_-EC/EMC solution. Figure 5 features a series of ED patterns after heating at 240 °C, 290 °C and 330 °C. The ED patterns in (a), (b) and (c) were obtained from NMC, while the ED patterns in (a’), (b’) and (c’) were obtained from LiPF_6_. In the ED patterns for NMC, a notable change could be detected at 330 °C. At 240 °C and 290 °C, the [122] and [201]-zone axis ED patterns from the *R*$\bar{3}$*m* layered structure were observed, as shown in Fig. 5(a,b). However, at 330 °C, streaks similar to those in Fig. 4(d) appeared, as indicated by the arrows in Fig. 5(c). In this case, likewise, the SEM observations showed that the size of NMC particles become smaller as a heating temperature increases (see Supplementary Fig. S2). In LiPF_6_, on the other hand, a noticeable change could be detected at 290 °C. At 240 °C, Debye–Scherrer rings were observable in addition to a halo pattern, as shown in Fig. 5(a’). Above 290 °C, a number of diffraction spots exhibiting strong intensity appeared, as indicated by the small arrows in Fig. 5(b’,c’). This temperature dependence of the ED patterns implied that the exothermic peaks at 255 °C and 306 °C in Fig. 3(b) could be attributed to the structural changes in LiPF_6_ and NMC, respectively.

**Figure 6:**
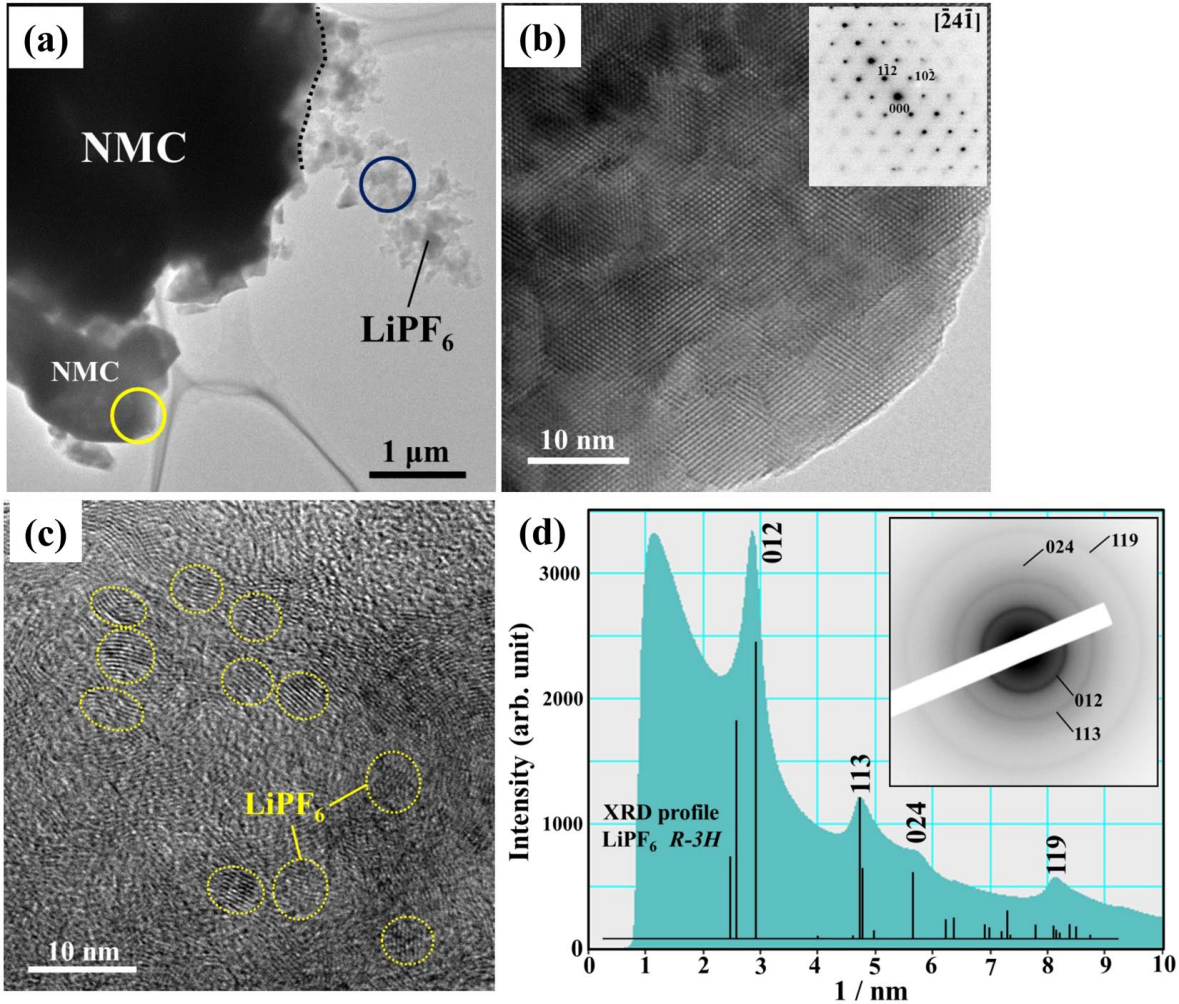
(**a**) The BF image showing the morphology of the charged NMC composite with the 1 M LiPF_6_-EC/EMC solution, which was heated at 240 °C. (**b**) The HR image taken from the NMC region indicated by the yellow circle in (**a**). (**c**) The HR image taken from the LiPF_6_ region indicated by the blue circle in (**a**). (**d**) The ED pattern obtained from the LiPF_6_ region and the corresponding intensity profile.

Figure 6 shows the *ex situ* TEM results for the charged NMC composite after heating at 240 °C. The BF image in Fig. 6(a) indicates the morphology of the charged NMC composite. The dark-contrast regions are related to NMC, while the other regions are related to LiPF_6_. The dotted line indicates the interface between NMC and LiPF_6_. It should be noted that the EC and EMC solvents were volatilized by heating in the DSC measurements. Figure 6(b,c) show the HR images displaying the microstructures of NMC and LiPF_6_, which were obtained from the regions indicated by the yellow and blue circles in Fig. 6(a), respectively. In Fig. 6(b), the lattice fringes corresponding to the $[\bar{2}4\bar{1}]$-zone axis ED patterns are clearly observable over the whole of the NMC particles. In Fig. 6(c), showing LiPF_6_, nanocrystallites with a size of approximately 5 nm are present in an amorphous matrix. Figure 6(d) shows the intensity profile of the ED pattern obtained from the LiPF_6_ region indicated by the blue circle in Fig. 6(a). Each intensity peak corresponds to that in the powder X-ray diffraction (XRD) pattern of LiPF_6_ with the space group *R*$\bar{3}$*H*. This suggests that the nanocrystallites observed in Fig. 6(c) can be identified as LiPF_6_. It is thus understood that no structural changes occurred over the temperature range from 20 °C to 240 °C.

**Figure 7:**
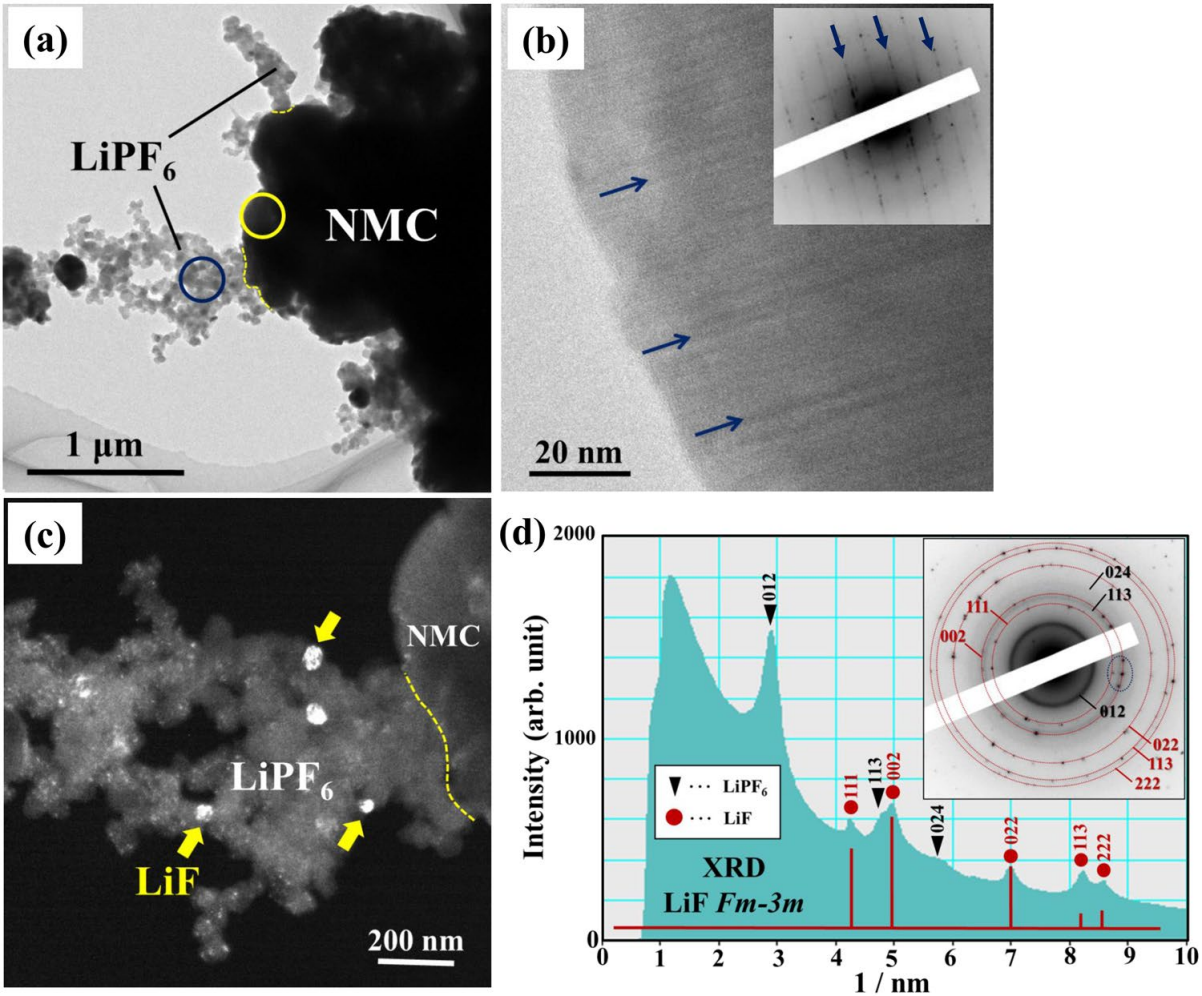
(**a**) The BF image showing the morphology of the charged NMC composite with the 1 M LiPF_6_-EC/EMC solution, which was heated at 330 °C. (**b**) The HR image taken from the NMC region indicated by the yellow circle in (**a**). (**c**) The DF image exhibiting the precipitation of LiF crystals in the LiPF_6_ region. (**d**) The ED pattern obtained from the LiPF_6_ region indicated by the blue circle in (**a**) and the corresponding intensity profile.

Figure 7 shows the *ex situ* TEM results for the charged NMC composite after heating at 330 °C. The BF image in (a) indicates the morphology of the charged NMC composite. The HR image (b) of NMC and the dark field (DF) image (c) of LiPF_6_ were taken from the regions indicated by the yellow and blue circles in (a), respectively. In Fig. 7(b), showing NMC, nanoscale linear contrasts indicating the presence of stacking faults are observed, as indicated by the arrows. The corresponding ED pattern in the inset exhibits a number of streaks similar to those shown in Fig. 4(d). Figure 7(d) shows the intensity profile of the ED patterns obtained from the LiPF_6_ region indicated by the blue circle in (a). In addition to the intensity peaks derived from LiPF_6_, the peaks corresponding to the XRD profile of the LiF crystalline phase with the space group *Fm*$\bar{3}$*m* appeared. Thus, the appearance of the Debye–Scherrer rings consisting of spots, which are detected in Fig. 5(b’,c’), is as a result of the precipitation of the LiF crystalline phase. The DF image in Fig. 7(c) was taken using the spots indicated by the dotted circles in the ED pattern shown in Fig. 7(d). It can be seen that LiF nanocrystallites with a size of approximately 50 nm are precipitated in the LiPF_6_ region, as indicated by the arrows in Fig. 7(c).

The *ex situ* TEM measurements reveal that significant structural changes occur in the charged NMC composites after heating at a temperature above the exothermic peaks. Firstly, the origin of the two exothermic reactions in the charged NMC composites with the 1 M LiPF_6_-EC/EMC solution is discussed. According to the TEM results, stacking faults developed in the layered *R*$\bar{3}$*m* structures of NMC in the temperature range of 290 °C to 330 °C, while LiF nanocrystallites precipitated in LiPF_6_ in the temperature range of 240 °C and 290 °C. With regard to the structural change in LiPF_6_, the appearance of LiF crystalline phase suggests that LiPF_6_ partially decomposes into LiF. This decomposition reaction is given by:

1 $${{\rm{LiPF}}}_{6}\to {\rm{LiF}}+{{\rm{PF}}}_{5}\,({\rm{gas}}).$$

Thus, the exothermic peak at 255 °C can be attributed to the heat generation accompanying the decomposition reaction (1). Furthermore, regarding to the structural change in NMC, as NMC become microparticlated, the *R*$\bar{3}$*m* layered structures collapse and stacking faults develop. According to previous studies, thermogravimetry and differential thermal analysis shows that weight loss occurs in delithiated LiCoO_2_ and NMC at above 200 °C, which is associated with oxygen loss^13,24,25^. It is thus anticipated that when stacking faults are introduced into the layered structures, the release of O_2_ gas would somewhat occur in NMC. In addition, some previous reports suggest that a release of oxygen arises from the particle surface by the appearance of *M*O-type rock salt crystal phases^26–29^. In the case of the present study, however, the surface of some NMC particles was found to be amorphized, as shown in Supplementary Fig. S3. Such an amorphous region was observed particularly in the charged NMC composite after heating at 330 °C. Because an amorphous region appears due to apparently the collapse of the NMC layered structure, a surface amorphous region of the particles would be also significantly involved in oxygen loss. Based on these, the exothermic peak at 306 °C is likely attributed to the heat generation accompanying the oxidation reaction between the released O_2_ gas and organic solvents. For example, the reaction between the EC solvent and O_2_ can be represented as follows:

2 $${({{\rm{CH}}}_{2}{\rm{O}})}_{2}{\rm{CO}}+5/2{{\rm{O}}}_{2}\to 3{{\rm{CO}}}_{2}+2{{\rm{H}}}_{2}{\rm{O}}.$$

Likewise, in the case of the charged NMC composite with only the EC/EMC solution, the exothermic reaction at 260 °C in Fig. 3(a) is also due to the oxidation reaction between the released O_2_ gas and organic solvents. However, the exothermic behavior resulting from the structural change in NMC is quite different depending upon the presence of LiPF_6_. The heating value of the exothermic peak at 306 °C in Fig. 3(b) is approximately 230 J g^−1^ lower than that at 260 °C, as seen in Fig. 3(a). Moreover, the former exothermic peak is shifted to a higher temperature by approximately 50 °C more than the latter. It is conceivable that precipitated LiF crystals may act as a coating layer, chemically stabilizing the surface of the NMC particles. This may contribute to the interference and suppression of the oxidation reaction between the released O_2_ gas and the organic EC/EMC solvents. The experimental results demonstrate that the structural changes in the electrolytes and cathode materials are directly associated with the exothermic reaction in the liquid electrolyte-based cells. To realize more favorable liquid electrolyte system, as a next step, electrochemical properties and thermal stability after more than 100 cycles will be investigated.

## Conclusion

The DSC measurements revealed that the charged NMC composite in liquid electrolyte-based cells exhibited exothermic reactions at approximately 255 °C and 306 °C. To clarify the origin of these exothermic reactions, in the present study, *ex situ* TEM measurements were conducted using samples after heating in the DSC measurements. In the sample that was heated to 290 °C, the LiPF_6_ electrolyte partially decomposed into LiF crystals. This suggested that the exothermic reaction at 255 °C could be attributed to the heat generation accompanying the decomposition reaction of the LiPF_6_. Furthermore, in the sample that was heated to 330 °C, a number of nanometer-sized stacking faults were introduced into the layered *R*$\bar{3}$*m* structures of NMC, inducing the release of O_2_ gas. Thus, the exothermic peak at 300 °C is derived from the oxidation reactions between the released O_2_ gas and the organic EC/EMC solvents. As a result, the experimental data reveals that structural changes in LiPF_6_ and NMC are directly responsible for the exothermic reactions present in the charged NMC composites in the liquid electrolyte-based lithium cells.

## Method

### Electrochemical measurements

The Li/LiNi_1/3_Mn_1/3_Co_1/3_O_2_ cells were charged and discharged at 25 °C with cut-off voltages of 3.0–4.4 V (vs. Li) using a charge-discharge measuring device (HJ1001SD8; Hokuto Denko Corp. or VSP; Bio-Logic Science Instruments). The current density of the initial charge-discharge cycle was measured at 0.2 mA cm^−2^. After the 2nd charge-discharge cycle, the current density was measured as 0.5 mA cm^−2^. CR2032 coin-type cells were used with a lithium metal sheet anode (16 mm in diameter, 0.5 mm in thickness). Li/Ni_1/3_Mn_1/3_Co_1/3_O_2_ (NMC, Nippon Chemical Industrial Co. Ltd), acetylene black (AB) and polyvinylidene difluoride (PVDF) were mixed to prepare the cathodes. The NMC electrodes were constructed by coating an Al sheet (15${\rm{\mu }}$m in thickness) with a mixture of NMC, AB and PVDF (85:5:10 wt.%), followed by drying and pressing. Cells were then fabricated using the NMC electrode (50 ${\rm{\mu }}$m in thickness containing an Al sheet) in the presence of the 1 M LiPF_6_ (EC/EMC = 70:30 vol.%) electrolyte. Alternate current (AC) impedance measurements were performed at 25 °C utilizing an impedance analyzer (VSP; Bio-Logic Science Instruments) in the frequency range from 10 mHz to 300 KHz. The excitation voltage for the measurements was 10 mV.

### Thermal stability tests

Samples for the DSC measurements (DSC, Rigaku Co., Thermo plus EVO2 8231) were prepared according to the following procedure. After charging, the cells were disassembled and the charged NMC electrodes were removed from the cells. Then, the electrodes were rinsed with EC/EMC (30:70 vol.%) and dried under vacuum at room temperature for 2 hours. Thereafter, the charged NMC composites (3 mg) with 1 M LiPF_6_ (EC/EMC = 30:70 vol.%) electrolytes (5 μL) or EC/EMC (30:70 vol.%) solvents (5 μL) were put together into a stainless-steel SUS pan for the DSC measurements, which was then crimp-sealed. The DSC measurements were carried out from room temperature to 330 °C with a heating rate of 5 °C min^−1^.

### TEM measurements

After the DSC measurements, *ex situ* TEM measurements were conducted at room temperature utilizing a JEM-2100F field-emission TEM system with an accelerating voltage of 200 kV. The vacuum in the TEM was approximately 1.0 × 10^−5^ Pa. To prevent the exposure of the samples to air, a vacuum transfer TEM holder (Gatan model 648) was used. The stainless-steel pan containing the charged NMC composites heated in the DSC measurements was disassembled in a glove box filled with dry argon gas using a vise. Then, the NMC composites were mounted on an amorphous carbon film supported by a Cu grid for the TEM measurements. The microstructures of the NMC composites were examined by taking the bright field (BF), dark field (DF), and high resolution (HR) TEM images and the corresponding electron diffraction (ED) patterns. A 14-bit charge-coupled device (CCD) camera was used as a recording medium to capture the images. Identification of the crystalline phases was conducted using computer program called “ProcessDiffraction”, in which the ED patterns of the polycrystalline and amorphous samples can be transformed into a one-dimensional intensity profile^30–33^.

## Electronic supplementary material

Supplementary information
