# Supplementary material for: Thermal behavior and microstructures of cathodes for liquid electrolyte-based lithium batteries
Source: Sci Rep. 2018 Oct 23;8:15613. doi: 10.1038/s41598-018-34017-2 (PMC6199343; doi:10.1038/s41598-018-34017-2)
Supplement: Supplementary file 1 — Supplementary information [file 41598_2018_34017_MOESM1_ESM.docx]

SUPPLEMENTARY INFORMATION TO

**Thermal behavior and microstructures of cathodes for liquid electrolyte-based lithium batteries**

Hirofumi Tsukasaki*, Wataru Fukuda, Hideyuki Morimoto, Toshihiro Arai, Shigeo Mori, Akitoshi Hayashi, Masahiro Tatsumisago

(*Corresponding Author)

**
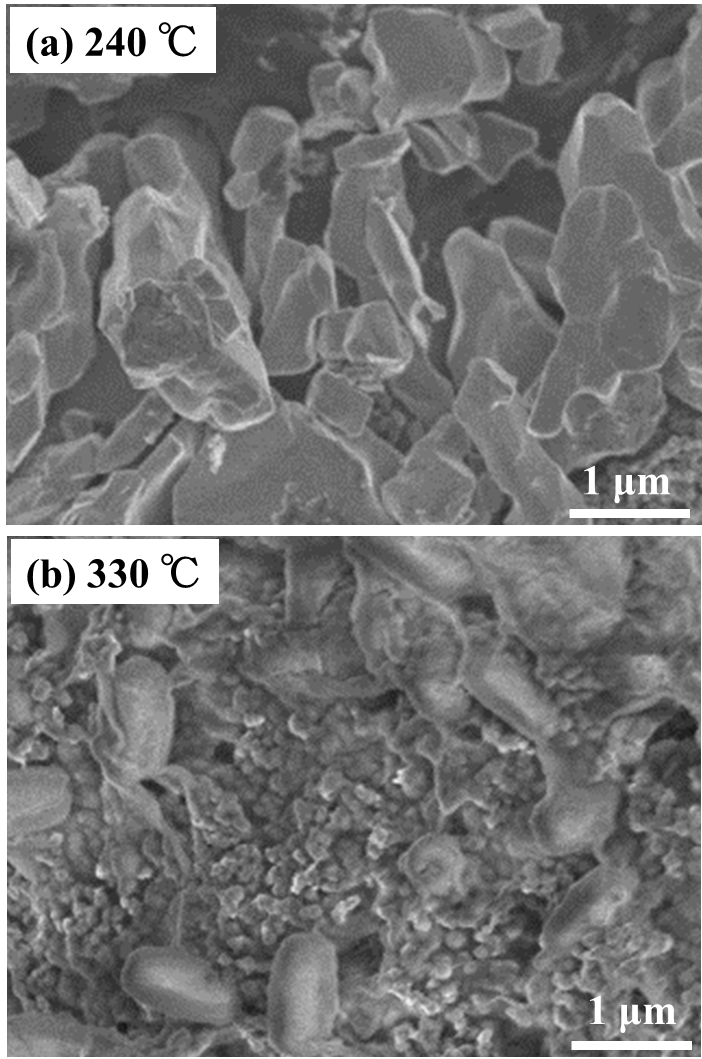
**

**Figure S1** shows the SEM images indicating the morphology of the NMC composites after the 2nd charge cycles in the presence of the EC/EMC solution. The image (a) and (b) were taken from the sample after heating at 240 °C and 330 °C, respectively. It can be seen that NMC particles after heating at 330 °C become finer than that after heating at 240 °C.

**
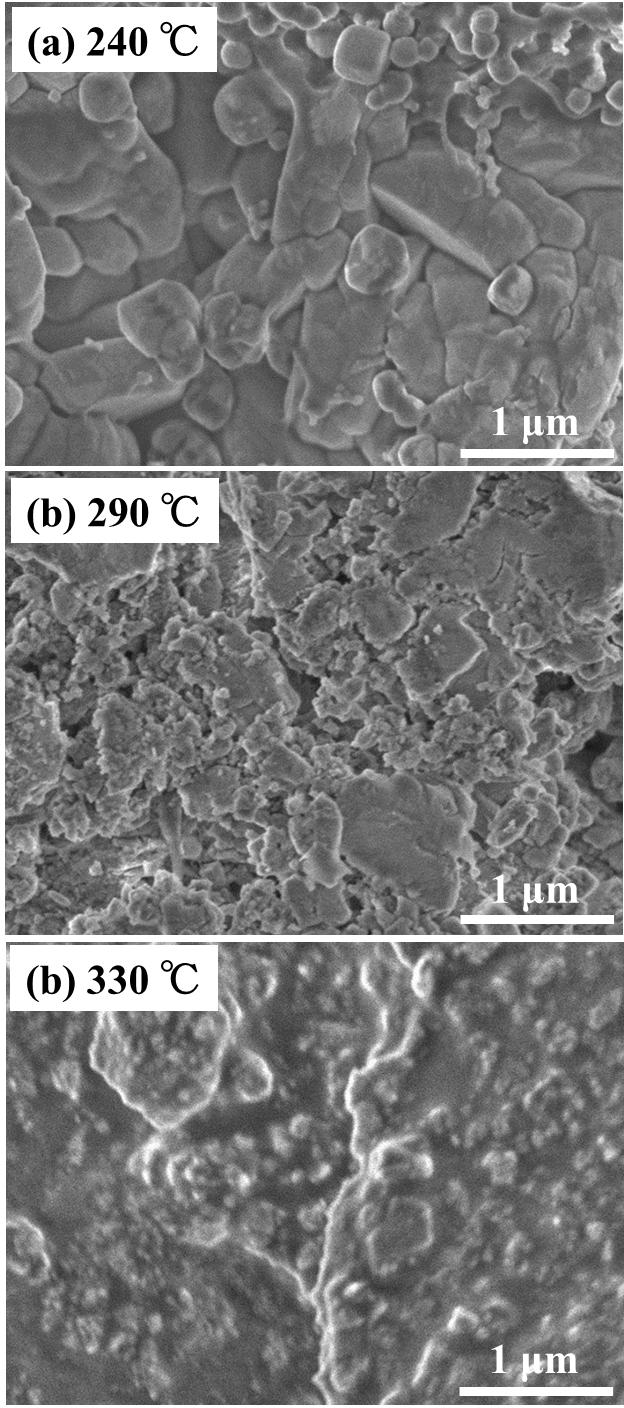
**

**Figure S2** shows the SEM images indicating the morphology of the NMC composites after the 2nd charge cycles in the presence of a 1 M LiPF_6_-EC/EMC solution. The image (a), (b) and (c) were taken from the sample after heating at 240 °C, 290 °C and 330 °C, respectively. It can be seen that the particle size of NMC become smaller as the temperature increases.


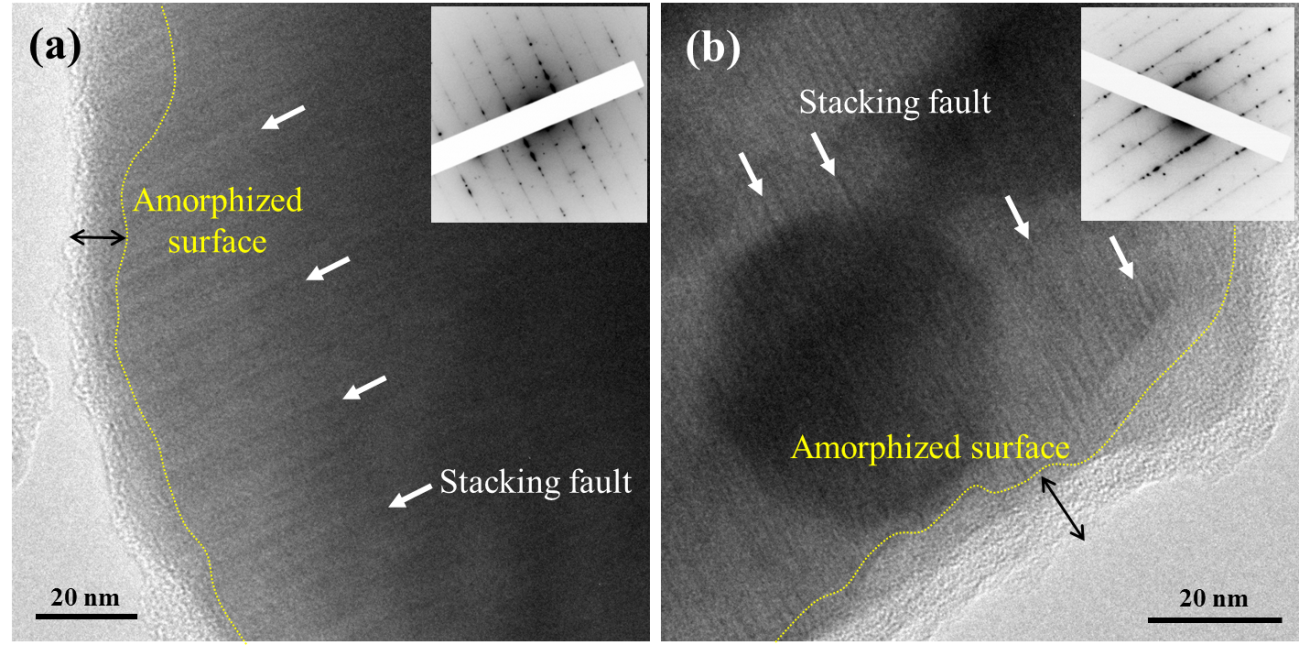


**Figure S3** shows the HR images indicating the microstructure of the NMC particles after heating at 330 °C. The HR images in (a) and (b) were taken from the charged NMC composites with EC/EMC and 1 M LiPF_6_-EC/EMC solutions, respectively. In both images, nanoscale linear contrasts indicating the presence of stacking faults were observed, as indicated by the white arrows. A number of streaks were also observable in the corresponding ED patterns in the inset. In addition, the edge of the NMC particles is amorphized, as indicated by the double arrows and dotted lines. It is likely that oxygen loss could occur from such an amorphized surface.
